# IL-2^high^ tissue-resident T cells in the human liver: Sentinels for hepatotropic infection

**DOI:** 10.1084/jem.20162115

**Published:** 2017-06-05

**Authors:** Laura J. Pallett, Jessica Davies, Emily J. Colbeck, Francis Robertson, Navjyot Hansi, Nicholas J.W. Easom, Alice R. Burton, Kerstin A. Stegmann, Anna Schurich, Leo Swadling, Upkar S. Gill, Victoria Male, TuVinh Luong, Amir Gander, Brian R. Davidson, Patrick T.F. Kennedy, Mala K. Maini

**Affiliations:** 1 Division of Infection and Immunity, Institute of Immunity and Transplantation, University College London, London, England, UK; 2 Centre for Digestive Diseases, Institute of Liver and Digestive Health, University College London, London, England, UK; 3 Centre for Immunobiology, Blizard Institute, Barts and The London School of Medicine and Dentistry, Queen Mary University of London, London, England, UK

## Abstract

The liver provides a tolerogenic immune niche exploited by several highly prevalent pathogens as well as by primary and metastatic tumors. We have sampled healthy and hepatitis B virus (HBV)–infected human livers to probe for a subset of T cells specialized to overcome local constraints and mediate immunity. We characterize a population of T-bet^lo^Eomes^lo^Blimp-1^hi^Hobit^lo^ T cells found within the intrahepatic but not the circulating memory CD8 T cell pool expressing liver-homing/retention markers (CD69^+^CD103^+^ CXCR6^+^CXCR3^+^). These tissue-resident memory T cells (T_RM_) are preferentially expanded in patients with partial immune control of HBV infection and can remain in the liver after the resolution of infection, including compartmentalized responses against epitopes within all major HBV proteins. Sequential IL-15 or antigen exposure followed by TGFβ induces liver-adapted T_RM_, including their signature high expression of exhaustion markers PD-1 and CD39. We suggest that these inhibitory molecules, together with paradoxically robust, rapid, cell-autonomous IL-2 and IFNγ production, equip liver CD8 T_RM_ to survive while exerting local noncytolytic hepatic immunosurveillance.

## Abstract (precis)

Pallett et al. identify tissue-resident memory CD8 T cells compartmentalized in the healthy human liver that expand in controlled hepatotropic infection and can swiftly produce antiviral cytokines. This prototype may inform the development of liver-targeted T cell immunotherapy.

## Introduction

The liver has a tolerogenic immunological landscape in keeping with its constant exposure to microbial products and food-derived antigens draining from the gut via the portal vein. Intrahepatic T cell responses must be regulated to protect this vital organ from excessive immunopathology (Protzer et al., 2012). The unique liver niche is frequently exploited by hepatotropic infections and tumors, which account for a huge burden of global mortality. For example, chronic hepatitis B (CHB) kills ∼780,000 people annually, and hepatocellular carcinoma (HCC) is the second leading cause of cancer deaths (GBD 2013 Mortality and Causes of Death Collaborators, 2015). There are currently intensive efforts to develop immunotherapeutic approaches for these liver diseases, stimulated by recent successes with other malignancies. The rationale for this goal is supported by the fact that most adults infected with hepatitis B virus (HBV) resolve the infection naturally, maintaining the virus under lifelong immune control. There is therefore an urgent need to characterize the features of T cells able to overcome tolerance in the liver to provide effective long-term immunosurveillance.

Little is known about the composition of the T cell compartment in the healthy human liver because of limitations in tissue access. It is critical to understand whether the liver contains specialized local populations capable of acting as sentinels against infection that cannot be studied by sampling blood. Recent studies in both mice and humans have revealed that a large proportion of memory CD8 T cells in nonlymphoid tissues are resident, representing functionally distinct populations of T cells poised to provide local protection against invading pathogens. Tissue-resident memory T cells (T_RM_) cannot reenter the circulation and are intimately adapted to individual organs by microenvironmental cues (Sathaliyawala et al., 2013; Schenkel and Masopust, 2014; Iijima and Iwasaki, 2015; Park and Kupper, 2015; Steinert et al., 2015; Thome and Farber, 2015; Fernandez-Ruiz et al., 2016; Hombrink et al., 2016; Mueller and Mackay, 2016). Elegant intravital imaging in mouse models has visualized CD8 T cells patrolling the extensive, narrow-lumenal, sinusoidal vasculature and surveying hepatocytes (through fenestra in the endothelium) for infection with HBV or malaria sporozoites (Guidotti et al., 2015; Fernandez-Ruiz et al., 2016). Limited data indicate that HBV-specific CD8 T cells are enriched in human livers (Maini et al., 2000; Fisicaro et al., 2010), but no studies have addressed whether these responses are simply an accumulation of the small populations that can be sampled in blood or whether they contain a discrete fraction sequestered in the liver.

In this study, we have analyzed T cells freshly isolated from the livers of a large number of healthy and HBV-infected donors, including unprecedented intrahepatic sampling from those with low viral loads or long-term resolution of HBV infection. We define the signature of a T_RM_ population within the human liver that cannot be sampled in the periphery, which is strikingly expanded in HBV infection, contains virus-specific responses and is associated with HBV control. The features that instruct these memory CD8 T cells to be retained, survive, and exert rapid noncytolytic antiviral cytokine production in the liver, in addition to the signals required for their induction, provide important insights for therapeutic vaccination and immunotherapy of CHB, HCC, and other hepatic diseases.

## Results and discussion

### A population of CD8 T cells expressing the tissue retention signals CD69/CD103/CXCR6 is sequestered in the healthy human liver

**Figure 1.**
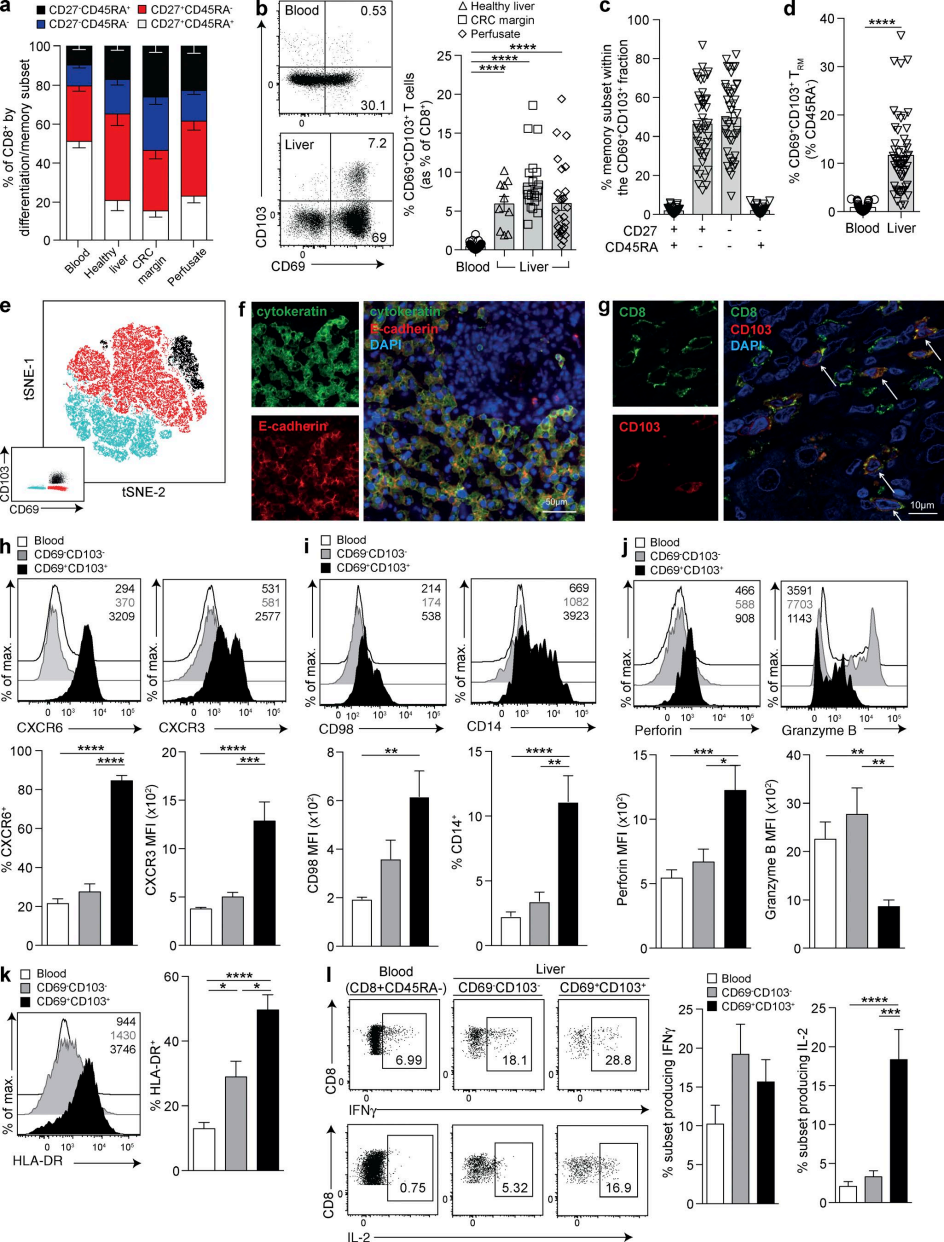
**Liver-resident memory CD8 T cells are present in the human liver but not in blood**. (a) Frequencies of CD8 T cell differentiation/memory subsets (CD27^+^CD45RA^+^; CD27^+^CD45RA^−^; CD27^−^CD45RA^−^; and CD27^−^CD45RA^+^) in peripheral blood (*n* = 40 healthy controls) and intrahepatic samples (*n* = 11 healthy liver tissues; *n* = 19 healthy margins of resected tissue distant to colorectal carcinoma metastases [CRC margins], and *n* = 24 perfusates). (b) Percent CD69^+^CD103^+^ of healthy control peripheral (*n* = 40) and intrahepatic CD8 T cells (*n* = 10 healthy liver tissues, *n* = 20 CRC margins, and *n* = 27 perfusates). (c) Intrahepatic CD69^+^CD103^+^ CD8 T cells stratified by memory phenotype in healthy livers (*n* = 47). (d) Percent CD69^+^CD103^+^ within memory (CD45RA^−^) CD8 in healthy control PBMCs (*n* = 40) or livers (*n* = 54). (e) The dimensionality reduction method tSNE analysis was used to generate a two-dimensional map of T cells within perfusate samples from healthy livers with regard to their expression of key transcription factors and tissue-residency markers. tSNE was performed on the expression data for the markers CD69, CD103, Blimp-1, Eomes, and T-bet as measured by FACS on all CD3^+^CD8^+^CD45RA^−^ events concatenated from four perfusates. Manual gating was used to identify CD69^+^CD103^+^ (black), CD69^+^CD103^−^ (red), and CD69^−^CD103^−^ T cells (blue) as shown in the bottom left corner. These gated populations were then plotted on to the total tSNE map. (f and g) Representative immunofluorescence staining of frozen liver sections: hepatocytes surrounding a vessel stained with cytokeratin (green) and e-cadherin (red; f), and another vessel stained with CD8 (green) and CD103 (red; g). Arrows in g denote cells coexpressing CD8 and CD103 in situ. (h–l) Representative examples and summary data from circulating (white), intrahepatic CD45RA^−^CD69^−^CD103^−^ (gray), and intrahepatic CD45RA^−^CD69^+^CD103^+^ (black) memory CD8 T cells from healthy donors for CXCR6 (%; *n* = 24) and CXCR3 (MFI; *n* = 21; h), CD98 (MFI; *n* = 12) and CD14 (%; *n* = 20; i), perforin (MFI; *n* = 16) and granzyme B (MFI; *n* = 16; j), HLA-DR (%; *n* = 21; k), and IFNγ and IL-2 intracellular cytokine production (4 h anti-CD3 and anti-CD28; *n* = 15; l). Error bars indicate means ± SEM; *, P < 0.05; **, P < 0.01; ***, P < 0.001; ****, P < 0.0001; p-values were determined via MANOVA (a); Kruskal-Wallis test (ANOVA) with a Dunn’s post hoc test for pairwise multiple comparisons (b, c, h, i, j, k, and l); and Mann-Whitney *t* test (d).

We performed an extensive study of the healthy human intrahepatic CD8 T cell compartment, with sixteen-parameter flow cytometric analysis (Fig. S1 a) of freshly isolated leukocytes from 54 liver samples (healthy liver pretransplantation biopsies or perfusates and resected healthy liver margins of colorectal metastases) compared with healthy donor PBMCs. The naive CD8 T cell compartment (CD27^+^CD45RA^+^) was significantly contracted in all liver samples compared with the periphery (P < 0.0001) and replaced with an expanded pool of memory subsets (CD27^+^CD45RA^−^, CD27^−^CD45RA^−^ and CD27^−^CD45RA^+^; Fig. 1 a).

To examine frequencies of CD8 T cells with the potential to be retained in the human liver, we analyzed their expression of CD69, a negative regulator of sphingosine 1 phosphate receptor 1 (*S1PR1*)–mediated T cell egress (Skon et al., 2013). Another bona fide tissue residency marker is the α chain of the integrin αEβ7, CD103 (Mackay et al., 2013; Sathaliyawala et al., 2013); the combination of CD103 with CD69 defined a population of cells within the intrahepatic CD8 T cell pool (Fig. 1 b) that was restricted to the CD45RA^−^ memory compartment (Fig. 1 c). CD69^+^CD103^+^ comprised a mean 11.4% of the intrahepatic memory pool, whereas they were virtually undetectable (mean <1%) in the periphery (Fig. 1 d), suggesting they were tissue-resident (henceforth referred to as T_RM_). In contrast, the CD69^−^CD103^−^ subset likely represented nonresident liver-infiltrating CD8 T cells (more frequent in blood than livers; Fig. S1 b); this is in line with the substantial population of memory T cells able to recirculate from the mouse liver (Steinert et al., 2015). The CD69^+^CD103^−^ subset was also greatly enriched in the liver and, like the CD69^+^CD103^+^ T_RM_, did not express CCR7 or CD62L but was not excluded from the periphery (Fig. S1, c and d).

In further support of CD69^+^CD103^+^ T_RM_ being a distinct subset, their transcription factor profile (T-bet^lo^Eomes^lo^Blimp-1^hi^Hobit^lo^; Fig. S1 e) was different from other liver and peripheral memory CD8 T cells. Their profile was similar to T_RM_ from other tissue sites (Hombrink et al., 2016; Mackay et al., 2016) with the exception of Hobit, which showed a converse pattern to mouse T_RM_ (Mackay et al., 2016) but analogous to human tonsillar T cells (Vieira Braga et al., 2015). Because liver T_RM_ could not be discriminated by a simple single or dual profile, we performed multidimensional analysis using t-distributed stochastic neighbor embedding (tSNE), confirming that their transcription factors contributed to CD69^+^CD103^+^ T_RM_, forming a discrete population (separated from the CD69^−^CD103^−^ by the intermediate CD69^+^CD103^−^ intrahepatic memory CD8 T cells; Fig. 1 e). Not only did CD69 and CD103 coexpression define discrete clusters of phenotypically similar cells on the tSNE map, but the transcription factors also showed clear clusters that overlapped with residency subsets (Fig. 1 e). The CD69^+^CD103^−^ subset was more heterogeneous in their transcription factor expression, suggesting they were composed of more than one discrete subset (Fig. S1 e). Further analysis revealed that this subset contained a large fraction of mucosal invariant T cells and a smaller proportion of γδ T cells, whereas CD69^+^CD103^+^ T_RM_ were largely conventional T cells (Fig. S1 f). We therefore focused our study primarily on comparing CD69^+^CD103^+^ and CD69^−^CD103^−^ liver subsets. However, future work could take advantage of the recently reported additional markers for tissue-residency of T cells in the mouse liver (McNamara et al., 2017) and human skin (Cheuk et al., 2017) to assess whether they can discriminate a population within the CD69^+^CD103^−^ liver subset restricted to conventional αβ T cells and excluded from the circulation.

The expression of CD103 on T_RM_ contributes to their retention in barrier tissues by allowing them to bind to epithelial cells expressing e-cadherin, the ligand for αEβ7 (Mackay et al., 2013). Healthy liver sections showed strong widespread e-cadherin staining of hepatocytes (Fig. 1 f), highlighting the potential relevance of this ligand for any liver T_RM_ within the parenchyma. Our findings suggest that CD103-expressing lymphocytes were frequently localized within the vasculature (because they were isolated from perfusates as well as tissue homogenates and visualized in vessels; Figs. 1 g and S1 g), from where they could potentially interact with e-cadherin–expressing hepatocytes through sinusoidal fenestra. This finding is analogous to the large fraction of T_RM_ noted to be in the marginated pool of mouse livers (Steinert et al., 2015).

Studies in mice have demonstrated the importance of CXCR6-dependent retention of memory CD8 T cells in the mouse liver for malaria protection, and human sinusoidal cells and hepatocytes can express the relevant ligand CXCL16 (Heydtmann et al., 2005; Wehr et al., 2013; Tse et al., 2014). T_RM_ (CD69^+^CD103^+^) and CD69^+^CD103^−^ CD8 T cells were markedly enriched for expression of the liver homing/retention receptor CXCR6 compared with CD69^−^CD103^−^ intrahepatic or peripheral memory CD8 T cells (Figs. 1 h and S1 h). We recently found that CXCR6 also marks a large subset of NK cells resident in the human liver (Stegmann et al., 2016). Human hepatic endothelium has also been shown to express CXCR3 ligands (Curbishley et al., 2005), and CXCR3 is critical for directing T_RM_ to virally infected mouse skin. Liver CD8 T_RM_ expressed much higher levels of CXCR3 than other intrahepatic and peripheral lymphocytes (Figs. 1 h and S1 h), suggesting that this chemokine receptor may also play a role in their homing and/or retention.

### Hepatic CD8 T_RM_ express high autocrine IL-2 and low granzyme B

Having identified a large population of CD8 T cells with a T_RM_ phenotype sequestered in the healthy human liver, we next investigated whether they were imprinted by their local milieu. To probe for evidence of the shaping of liver T_RM_ by their nutrient microenvironment, we assessed their expression of system-L amino acid transporters marked by CD98. The liver T_RM_ fraction was selectively enriched for CD98 expression (Figs. 1 i and S1 i), which we have shown to be up-regulated in response to arginine deprivation (Pallett et al., 2015). Intriguingly, a proportion of liver T_RM_ expressed CD14, the coreceptor for TLR-4–mediated LPS recognition, that was barely expressed on CD69^−^CD103^−^ or circulating memory CD8 T cells (Figs. 1 i and S1 i). CD14 has previously been reported to be induced on T cells by TCR engagement and cytokine stimulation, rendering them responsive to LPS, with the production of high concentrations of IFNγ (Komai-Koma et al., 2009). Further work is required to investigate whether CD14 on liver T_RM_ can regulate responsiveness to the large amounts of LPS that they would encounter from the portal blood supply.

To assess the functional adaptations of liver CD8 T_RM_, we stained them directly ex vivo for cytotoxic mediators. Although they expressed more perforin than their CD69^−^CD103^−^ intrahepatic and circulating counterparts, hepatic CD8 T_RM_ had markedly reduced levels of the serine protease granzyme B (Fig. 1 j) and less granzyme A and K (Fig. S1 j). This suggested the possibility of down-regulated capacity for immediate cytotoxicity, limiting T cell–mediated immunopathology in the healthy liver. Despite their selectively impaired granzyme expression, CD8 T_RM_ had increased levels of ex vivo activation (HLA-DR) compared with the CD69^−^CD103^−^ intrahepatic or circulating memory CD8 T cells (Fig. 1 k). Upon short-term polyclonal stimulation to assess their immediate effector potential, liver CD8 T_RM_ were able to produce equivalent amounts of IFNγ to the CD69^−^CD103^−^ fraction. Most strikingly, a much larger proportion of liver CD8 T_RM_ produced IL-2 compared with their CD69^−^CD103^−^ and circulating counterparts. A mean of 23.5% (maximum 65.2%) of intrahepatic CD8 T_RM_ produced IL-2 after just 4 h of stimulation, whereas there was negligible production by circulating memory CD8 T cells (Fig. 1 l). This unusually high IL-2 production is likely to be critical to the protective potential of hepatic CD8 T_RM_ as it has been demonstrated that CD8 T cells need to make their own cell-autonomous supplies of IL-2 to drive adequate IFNγ production and persistence of memory responses to pathogens (Zimmerli et al., 2005; Feau et al., 2011). The capacity of CD8 T cells to produce and consume their own IL-2 may be particularly relevant for the maintenance of effective memory responses in peripheral tissues like the liver, where CD4 T cell frequencies are low, and priming of CD4 T cell responses is suboptimal (Zimmerli et al., 2005; Crispe, 2014).

### Liver T_RM_ are a tightly regulated population that expand in hepatotropic viral infection

**Figure 2.**
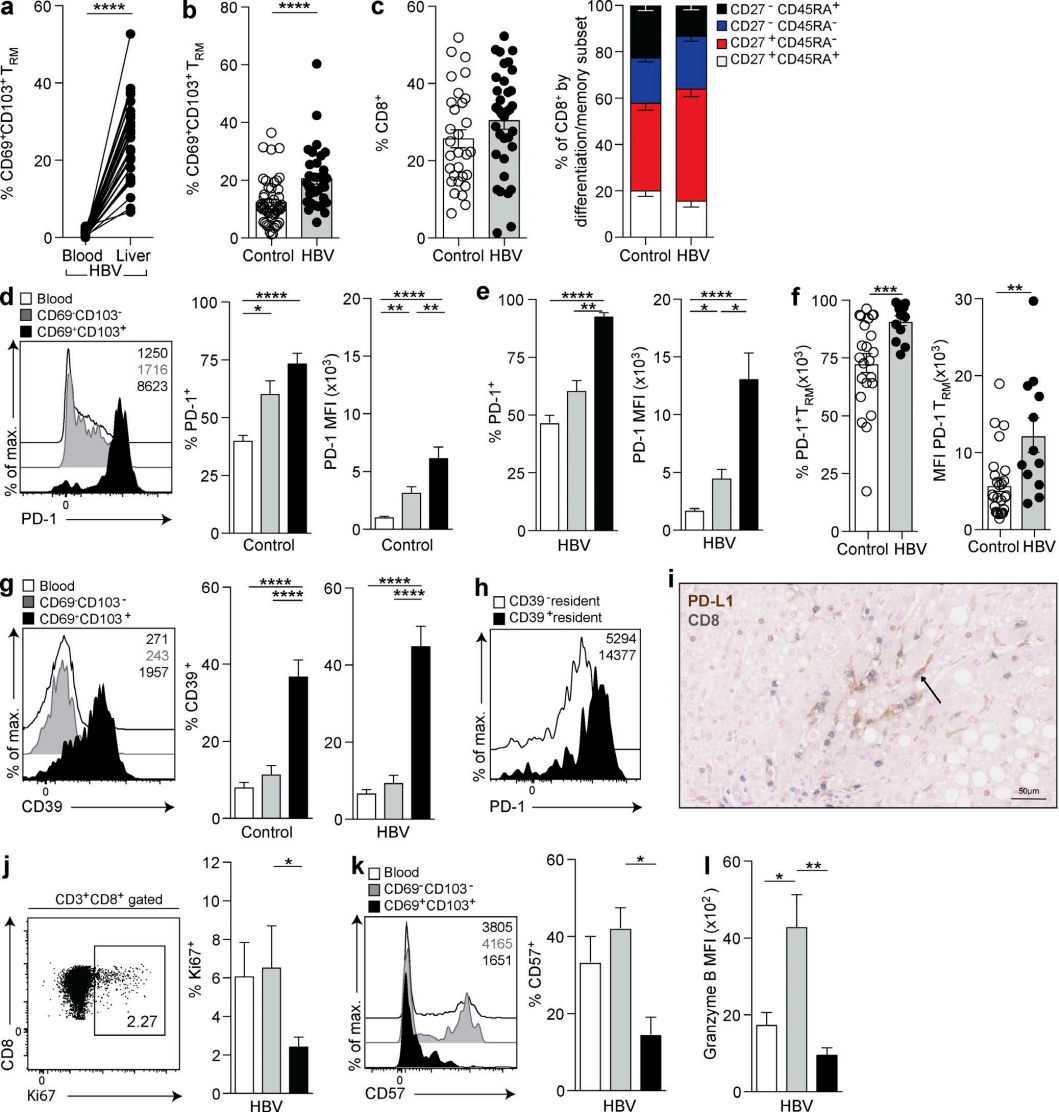
**CD8 T_RM_ expand in patients controlling HBV infection.** (a and b) Frequencies of CD69^+^CD103^+^ as percentages of the CD8 T cell memory (CD45RA^−^) pool in paired blood and liver biopsies from patients with chronic HBV infection (*n* = 33; a) and biopsy/resection tissue from healthy (*n* = 54) or HBV-infected (*n* = 35) livers (b). (c) Percent intrahepatic CD8 T cells within live singlet lymphocytes and stratified by differentiation/memory subsets (defined in Fig. 1). (d–f) PD-1 expression (percentage and MFI) on circulating (white), intrahepatic CD45RA^−^CD69^−^CD103^−^ (gray), and intrahepatic CD45RA^−^CD69^+^CD103^+^ (black) memory CD8 T cells isolated from healthy controls (*n* = 26; d), patients with CHB (*n* = 13; e), and comparing intrahepatic CD45RA^−^CD69^+^CD103^+^ CD8 T cells from healthy controls and CHB (f). (g) CD39 expression (percentage) on circulating (white), intrahepatic CD45RA^−^CD69^−^CD103^−^ (gray), and intrahepatic CD45RA^−^CD69^+^CD103^+^ (black) memory CD8 T cells isolated from healthy controls (*n* = 25) or CHB (*n* = 18). (h) Representative example of PD-1 expression on CD39^−^/CD39^+^ CD45RA^−^CD69^+^CD103^+^ CD8 T cells. (i) Representative immunohistochemical staining of PD-L1 (brown) and CD8 (gray) using a paraffin-embedded liver section. Arrow denotes PD-L1 staining on a cell localized in the liver sinusoids. (j–l) Ki67 (%; *n* = 10; j), CD57 (%; *n* = 5; k) and granzyme B (MFI; *n* = 9; l) expression on circulating (white), intrahepatic CD45RA^−^CD69^−^CD103^−^ (gray), and intrahepatic CD45RA^−^CD69^+^CD103^+^ (black) on memory CD8 T cells from patients with CHB. Error bars indicate means ± SEM; *, P < 0.05; **, P < 0.01; ***, P < 0.001; ****, P < 0.0001; p-values were determined via Wilcoxon Signed-rank *t* test (a); Mann-Whitney *t* test (b, c, and f); MANOVA (c); and Kruskal-Wallis test (ANOVA) with a Dunn’s post hoc test for pairwise multiple comparisons (c, d, e, g, j, k, and l).

To examine the relevance of T_RM_ to local immune defense in the liver, we investigated their in vivo response to persistent infection with the highly prevalent hepatotropic virus HBV. We obtained paired blood and surplus diagnostic liver biopsy samples from 33 patients with treatment-naive CHB; in every case, CD8 T_RM_ were detectable in the liver and remained excluded from the blood (Fig. 2 a), as in healthy controls. Intrahepatic CD8 T_RM_ were highly enriched in the setting of HBV infection compared with healthy livers, with a mean threefold increase in frequency, accounting for up to 68.5% of all intrahepatic memory CD8 T cells (Fig. 2 b). This reflected an increased conversion to a resident phenotype within the pool of intrahepatic CD8 T cells rather than selective expansion of the T_RM_ subset because there was no significant change in the overall proportion of CD8 T cells or memory subsets in an HBV-infected liver relative to a healthy liver (Fig. 2 c).

The liver contains a large proportion of CD8 T cells compared with the blood (mean 72% versus 31% of total CD3 T cells in this cohort); these must be tightly regulated to prevent immunopathological damage. The coinhibitory receptor PD-1 is known to play a central role in liver tolerance in mouse models (Iwai et al., 2003; Isogawa et al., 2005) and in constraining intrahepatic antiviral responses to HBV (Fisicaro et al., 2010), but little is known about its expression on T cell subsets within the healthy human liver. The percentage of memory CD8 T cells expressing PD-1 and the level of its expression (mean fluorescence intensity; MFI) showed a stepwise increase from blood to nonresident to resident populations in healthy livers (Fig. 2 d). PD-1 was significantly higher on T_RM_ within HBV-infected livers than on nonresident or circulating memory CD8 T cell counterparts in these patients (Fig. 2 e) and compared with levels on T_RM_ in healthy controls (Fig. 2 f). Virtually all CD8 T_RM_ in HBV-infected livers expressed PD-1 (mean 92.4%), and their level of PD-1 expression (MFI) was more than twofold higher than on nonresident CD8 T cells (Fig. 2 e) or T_RM_ in healthy livers (Fig. 2 f).

The ectonucleotidase CD39 was also expressed on a much higher percentage of CD8 T_RM_ in healthy and HBV-infected livers compared with nonresident intrahepatic and circulating memory CD8 T cells (Fig. 2 g). CD39 has recently been found to identify terminally exhausted CD8 T cells expressing the highest level of PD-1 within virus-specific populations circulating in patients with hepatitis C and in mice with lymphocytic choriomeningitis virus (Gupta et al., 2015). In line with this finding, CD39 marked CD8 T_RM_ with the highest PD-1 expression in the liver (Fig. 2 h). Our data reveal that CD39 expression is a feature of a large proportion of liver-resident CD8 T cells. The high-level expression of the exhaustion markers PD-1 and CD39 on liver CD8 T_RM_ was at odds with their capacity to mount immediate strong cytokine responses (e.g., IL-2–producing cells expressed equivalently high PD-1 and CD39 to the rest of T_RM_; Fig. S2 a). Their efficient production of cell-autonomous IL-2 may be instrumental in instructing this “poised” state. In support of this, IL-2 production by mouse CD8 T cells allows them to overcome PD-L1–mediated inhibition by liver sinusoidal endothelial cells (Schurich et al., 2010).

PD-L1 can be expressed by several liver-resident cell types and further up-regulated in inflammatory settings such as viral hepatitis (Iwai et al., 2003; Mühlbauer et al., 2006). In support of this, we observed strong PD-L1 staining on sinusoidal populations with morphology suggestive of Kupffer cells, in close proximity to CD8 T cells in CHB (Fig. 2 i). Recent work using genetic knockdown of PD-1 on mouse CD8 T cells has pointed to a central role for this coinhibitory receptor in preserving T cells in the face of chronic antigenic stimulation by preventing excessive proliferation and terminal senescence (Odorizzi et al., 2015). Congruent with CD8 T_RM_ expressing high PD-1 in an environment enriched for PD-L1 expression, direct ex vivo staining confirmed constraints on their proliferation (Ki67; Fig. 2 j), senescence (CD57; Figs. 2 k and S2 b), and cytotoxicity (granzyme B; Fig. 2 l).

### Sequential exposure to IL-15 and TGFβ and/or TCR engagement induces the PD-1^hi^ liver-resident CD8 T cell phenotype

**Figure 3.**
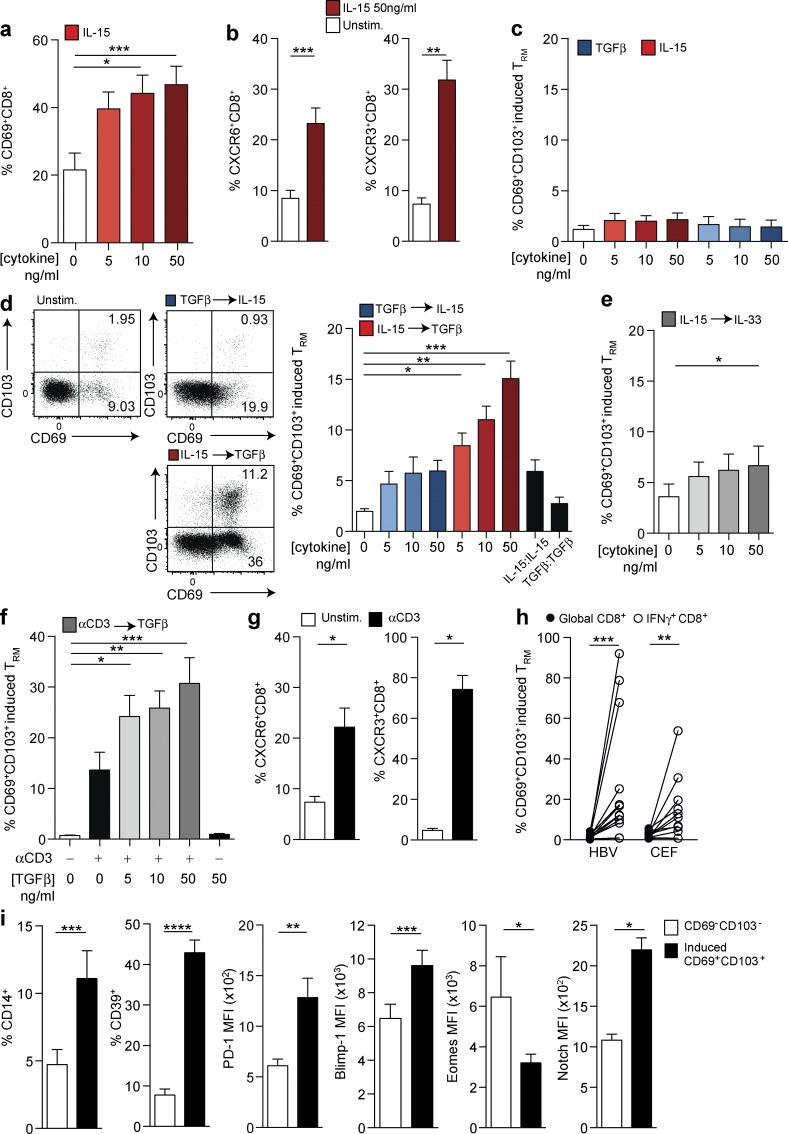
**Sequential exposure to IL-15 or antigen and TGFβ induces CD8 “residency” in vitro**. (a and b) CD69 expression (%; *n* = 27; a) and CXCR6 (%; *n* = 17) or CXCR3 (%; *n* = 12; b) expressing peripheral CD8 T cells after 3 d culture in the presence of rhIL-15 at indicated doses (red; *n* = 11). (c and d) Coexpression of CD69 and CD103 on peripheral CD8 T cells (“induced” CD69^+^CD103^+^ CD8 T cells; *n* = 11) after either rhIL-15 alone (red) or rhTGFβ alone (blue; c) at the indicated doses for 6 d, two-step sequential cytokine exposure with 3 d rhIL-15 followed by a further 3 d rhTGFβ (red) at concentrations of both cytokines indicated or vice versa (blue; *n* = 20; d) or two-step sequential cytokine exposure with 3 d rhIL-15 followed by 3 d rhIL-33 at concentrations of both cytokines indicated (*n* = 11; e). (f) Frequency of induced CD69^+^CD103^+^ CD8 T cells after two-step culture with 3 d 0.25 µg/ml immobilized anti-CD3 followed by 3 d ± addition of rhTGFβ at concentrations indicted (*n* = 12). (g) Expression of CXCR6 (%; *n* = 12) and CXCR3 (%; *n* = 12) on peripheral CD8 T cells after 6 d 0.25 µg/ml immobilized anti-CD3 (*n* = 10). (h) Frequency of induced CD69^+^CD103^+^ within the global or peptide-specific (HBV or CEF) CD8 T cells after culture with either 1 µg/ml of overlapping peptides spanning the core region of HBV genotype D or 0.5 µg/ml CEF (peptide pool against CEF) for 7 d (*n* = 12). (i) Expression of CD14 (%; *n* = 17), CD39 (%; *n* = 20), PD-1 (MFI; *n* = 17), Blimp-1 (MFI; *n* = 12), Eomes (MFI; *n* = 6), and Notch (MFI; *n* = 6) on CD69^−^CD103^−^ CD8^+^ (white) and CD69^+^CD103^+^ (“induced resident;” black) CD8 T cells after sequential exposure to 3 d of 50 ng/ml rhIL-15 followed by 3 d of 50 ng/ml rhTGFβ. All figures show summary data from at least four independent experiments. Error bars indicate means ± SEM; *, P < 0.05; **, P < 0.01; ***, P < 0.001; ****, P < 0.0001; p-values were determined via a Kruskal-Wallis test (ANOVA) with a Dunn’s post hoc test for pairwise multiple comparisons (a, c, d, e, and f) or a Wilcoxon Signed-rank *t* test (b, g, h, and i).

We next investigated the signals required to impose or maintain the liver residency phenotype on CD8 T cells. PBMCs devoid of CD8 T_RM_ were treated with cytokines relevant to the liver and/or the promotion of tissue residency (IL-15, TGFβ, IL-33, IL-7, IL-12, TNF, CXCL16, and IL-6). Of the cytokines tested, IL-15 was able to induce CD69 expression on peripheral CD8 T cells in a dose-dependent manner (Fig. 3 a) as well as up-regulating the chemokine receptors CXCR6 and CXCR3 (Fig. 3 b) that we previously noted on ex vivo liver T_RM_. IL-15 is constitutively produced in the liver (Golden-Mason et al., 2004), is capable of inducing antigen-independent T cell differentiation (Alves et al., 2003), and drives bystander T cell activation in viral infections such as HBV (Sandalova et al., 2010). None of the cytokines alone, including IL-15 or TGFβ, were able to induce significant expression of CD103, required to tether CD8 T cells to e-cadherin for their retention in the liver (Fig. 3 c). However, sequential exposure to IL-15 followed by TGFβ efficiently induced de novo CD8 T_RM_ expressing both CD69 and CD103 at a similar frequency to that found in the healthy liver (Fig. 3 d). Reversal of the two-step cytokine signal (TGFβ followed by IL-15) did not induce T_RM_ (Fig. 3 d), whereas IL-15 followed by IL-33 (equivalent to TGFβ in its capacity to induce tissue residency in mice; Mackay et al., 2013) was only able to induce marginal expansion of human CD69^+^CD103^+^ CD8 T cells (Fig. 3 e).

The induction of tissue residency can also be instructed by local antigen in mouse viral infection or vaccination (Fernandez-Ruiz et al., 2016; Khan et al., 2016; Muschaweckh et al., 2016). To test this possibility, we substituted IL-15 with a TCR-dependent signal initially by cross-linking CD3. TCR stimulation, similar to IL-15, showed a potent capacity to recapitulate the liver CD8 T_RM_ phenotype using human PBMCs, with induction of a population expressing CD69 and CD103 and up-regulation of CXCR6 and CXCR3 (Fig. 3, f and g). The TCR-mediated induction of CD8 T cells expressing CD69 and CD103 was further enhanced by subsequent stimulation with TGFβ in a dose-dependent manner (Fig. 3 f). T_RM_ could be induced from antigen-experienced (CD45RA^−^) and naive (CD27^+^CD45RA^+^) peripheral CD8 T cells (more readily from the former; Fig. S2 c), suggesting that both fractions may be amenable to therapeutic attempts to enhance tissue residency. To examine whether recognition of HBV-infected hepatocytes could contribute to the increased expansion of liver CD8 T_RM_ we had observed in patients with CHB, we used overlapping peptides spanning the HBV core region (or peptides representing control epitopes from cytomegalovirus/Epstein–Barr virus/influenza; CEF) as the TCR stimulus. HBV and other viral peptide-responding (IFNγ^+^) CD8 T cells up-regulated both CD69 and CD103 (Fig. 3 h), indicating that antigen recognition can substitute for cytokines for in situ induction of the liver T_RM_ phenotype.

We went on to examine CD8 T_RM_ induced from PBMCs (by sequential IL-15/TGFβ or TCR engagement/TGFβ) for expression of other markers we had found to characterize T_RM_ extracted from human livers. We found that CD8 T cells with an in vitro “induced residency” phenotype also developed a CD14^+^CD39^+^PD-1^hi^ and Blimp^hi^Eomes^lo^ profile (Fig. 3 i) comparable to the phenotypic and transcriptional adaptations characterizing liver-resident populations. In addition, they up-regulated Notch (Fig. 3 i), which drives a program of human lung residency (Hombrink et al., 2016), underscoring some striking similarities between T_RM_ in different human organs (Hombrink et al., 2016; Wong et al., 2016). Our data delineate signals relevant to the liver environment that are capable of recapitulating the phenotype of liver-resident CD8 T cells. We show that TGFβ, a prototypic liver cytokine, plays a key role in inducing CD103 expression to retain CD8 T cells that have received an initial homing signal from the bystander proinflammatory cytokine IL-15. Kupffer cells, specialized macrophages that line hepatic vasculature, constitute a major source of TGFβ and IL-15 as well as the ligands for CXCR6 and PD-1 and are therefore likely candidates to imprint infiltrating T cells with signals driving the combination of features allowing them to reside and survive in the liver microenvironment. IL-15 and TGFβ are together capable of inducing a population of resident T cells while also imposing a highly constrained (PD-1^hi^CD39^+^) phenotype to facilitate their long-term survival in the face of antigenic challenge. TCR engagement, for example by in situ recognition of viral epitopes, can substitute for these cytokine signals or can synergize with TGFβ to further promote T cell residency in the human liver, likely contributing to the increased T_RM_ observed in HBV infection. Our data suggest that a TCR signal or the bystander cytokine IL-15 should precede exposure to TGFβ for optimal induction of CD69/CD103 coexpressing T_RM_.

### Liver-resident CD8 T cells containing IL-2^high^ multispecific HBV-specific T cells are associated with viral control

**Figure 4.**
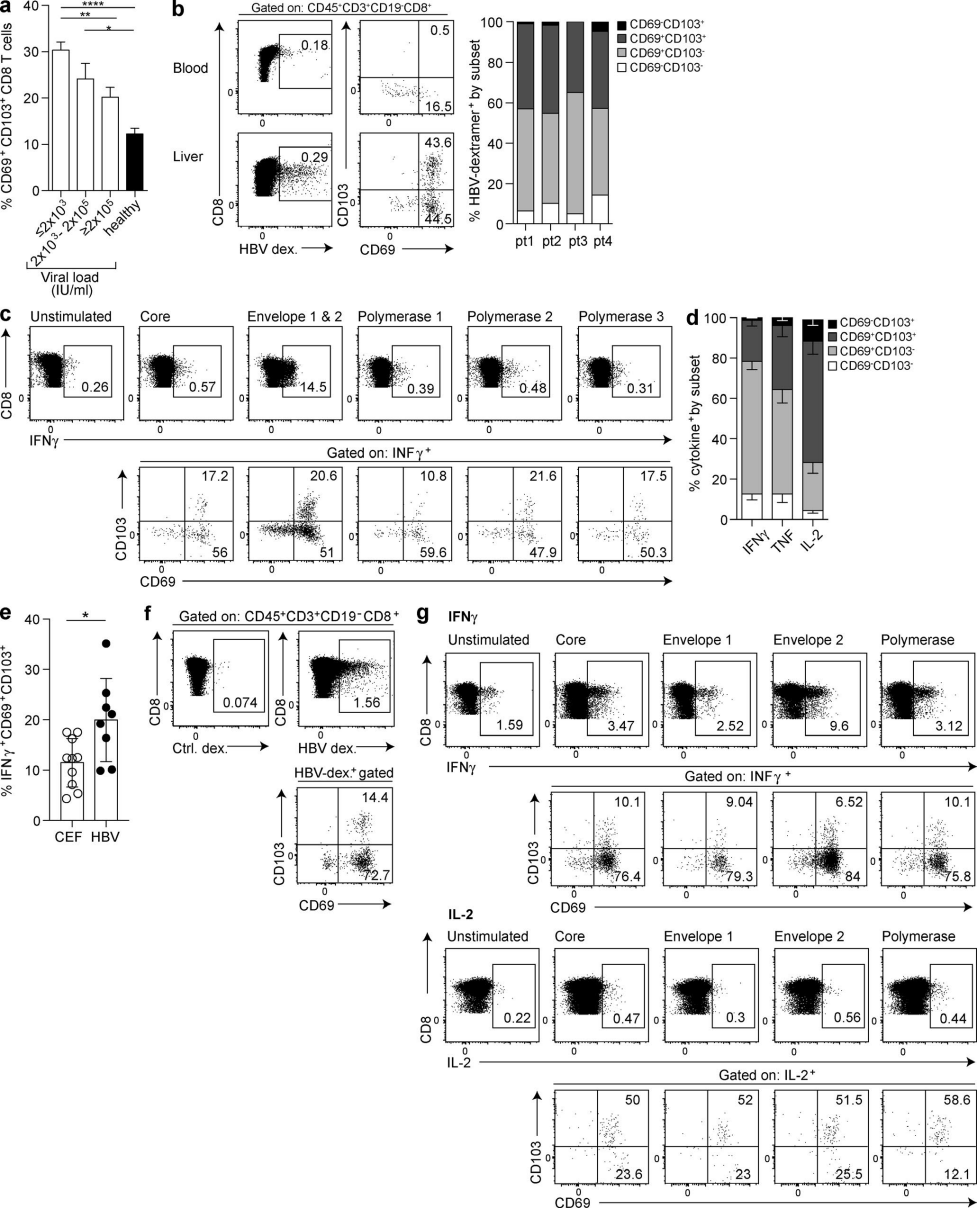
**Liver CD8 T_RM_ include virus-specific CD8 and are associated with HBV control.** (a) Frequencies of CD45RA^−^CD69^+^CD103^+^ CD8 T cells in HBV-infected liver biopsies stratified by viral load (IU/ml; ≤2,000, *n* = 10; 2 × 10^3^ − 2 × 10^5^, *n* = 15; >2 × 10^5^, *n* = 8; all treatment naive) compared with healthy livers (*n* = 54). (b–d) Proportion of HBV-specific CD8 T cells in CHB expressing residency markers CD103 and CD69, identified by ex vivo staining with a panel of HLA-A2–HBV peptide dextramers (example plots for blood and an HBV^+^ resection liver; summary data *n* = four livers; one resection, three biopsies; b) or after stimulation of IHL for 16 h with 10 µg/ml of overlapping peptides spanning core, polymerase, or envelope regions (using an HBsAg^+^ HBV perfusate; c) and after 16-h stimulation with core (HBV genotype D) alone and intracellular cytokine staining for IFNγ, TNF, and IL-2 (*n* = 7; d). (e) Proportion of CEF–specific (*n* = 10; four margins, six biopsies) and HBV-specific (*n* = 8; one resection, one perfusate, six biopsies) IFNγ CD8 responses expressing a CD45RA^−^CD69^+^CD103^+^ phenotype. (f) Detection of CD8 T cells according to CD69 and CD103 expression within ex vivo HLA-A2–HBV dextramer (dex.) panel-binding cells in an HLA-A2^+^ perfusate sample from an individual with HBsAg-resolved HBV infection. (g) IHLs from an HLA-A2 perfusate from an individual with HBsAg-resolved HBV infection were stimulated with 10 µg/ml of overlapping peptides spanning core, envelope, or polymerase regions of HBV genotype D for 16 h followed by intracellular cytokine staining for IFNγ and IL-2 and assessment of residency markers on cytokine-positive populations. Error bars indicate means ± SEM; *, P < 0.05; **, P < 0.01; ****, P < 0.0001; p-values were determined by a Kruskal-Wallis test (ANOVA) with a Dunn’s post hoc test for pairwise multiple comparisons (a) or Wilcoxon Signed-rank *t* test (e).

We next took advantage of the heterogeneity of disease outcome after infection with HBV to probe the protective potential of T_RM_ in the human liver. We observed that the HBV viral load showed a stepwise decrease as the frequency of intrahepatic CD8 T_RM_ increased, with the highest frequency found in patients with well-controlled infection (≤2,000 IU/ml; Figs. 4 a and S3 a), whereas there was no correlation with levels of surface antigen (hepatitis B surface antigen; HBsAg), liver inflammation (alanine transaminase) or hepatitis B ‘e’ antigen (HBeAg; Fig. S3 a). Congruent with their association with viral control, the T_RM_ pool contained HBV-specific CD8 T cells identified by staining with a combined panel of HLA-A2–peptide dextramers (in a subset of four HLA-A2^+^ individuals; Fig. 4 b). Around 40% of the dextramer-stained HBV-specific CD8 T cells in the liver were classical T_RM_ (CD45RA^−^CD69^+^CD103^+^), whereas another large fraction (>40%) were within the CD69^+^CD103^−^ subset (Fig. 4 b) that is also largely compartmentalized within the liver. Thus, a major proportion of HBV-specific CD8 T cells are likely resident in the liver, implying that the small subset sampled in the periphery is not representative of the whole population.

To assess the effector potential and multispecificity of intrahepatic virus-specific CD8 T cells, we obtained perfusates from HBV-infected livers; these yielded sufficient lymphocytes to allow overnight stimulation with individual pools of peptides spanning different HBV proteins (which did not alter their CD69/CD103 phenotype; Fig. S3 b), before intracellular cytokine staining. This gave the first ex vivo insight into the multispecificity of the intrahepatic HBV-specific response, revealing the presence of CD8 T cells responding to each of the HBV protein pools, with a particularly large response to the envelope peptides (example in Fig. 4 c). This contrasts with the much lower frequency of circulating HBV-specific CD8 T cells, and in particular of envelope-specific CD8 T cells, found in previous peptide stimulation studies using PBMCs (Bertoletti and Ferrari, 2016). Consistent with extensive compartmentalization of the HBV-specific response at the site of infection, the majority of intrahepatic cytokine-producing CD8 T cells had either a CD69^+^CD103^−^ or a CD69^+^CD103^+^ phenotype (Fig. 4, c and d), congruent with ex vivo and cytokine-negative CD8 (Fig. S3 b). Intrahepatic CD8 T cells with a T_RM_ phenotype were able to produce IFNγ and TNF upon recognition of HBV epitopes thathave been shown to mediate noncytolytic clearance of HBV from infected hepatocytes. Whereas IL-2–producing HBV-specific CD8 T cells are virtually undetectable in PBMC studies of CHB (Boni et al., 2012), IL-2 production was strikingly enriched in the HBV-specific T_RM_ compartment (Fig. 4 d). Comparison of intrahepatic responses against HBV and control (CEF) peptides revealed that some intrahepatic CEF responses also had a CD69^+^CD103^+^ phenotype, but this liver residency phenotype was increased within HBV-specific CD8 (Fig. 4 e).

To further investigate our postulate that IL-2 production is a key feature of liver T_RM_ allowing their long-term survival and local antiviral efficacy, we took advantage of unique access to intrahepatic lymphocytes (IHLs) from two donors who had previously achieved immune resolution of their HBV infection. Such donors (HBsAg^−^, anti-HBsAb^+^/anti-HBcAb^+^, and HBV DNA undetectable), who have previously only been studied in the periphery, provide a model for “functional cure” of HBV because traces of virus remain under active control by persistent T cell responses and can reactivate during immunosuppressive therapy (Rehermann et al., 1996). Investigation of these “gold-standard” responses at the site of immune control showed the persistence of virus-specific T cell responses in the liver after disease resolution; these were detectable directly ex vivo (stained with a panel of HLA-A2/peptide multimers; Fig. 4 f) and directed against multiple HBV proteins, particularly envelope (after overnight peptide stimulation; Fig. 4 g). Around 10% of these intrahepatic IFNγ^+^ responses were CD69^+^CD103^+^, and the majority of the remainder were CD69^+^CD103^−^ (similar to ex vivo and IFNγ^−^ CD8; Fig. S3 b), suggesting that they may be able to reside in the liver to prevent reactivation of residual intrahepatic HBV. Responses against all peptide pools produced IL-2, and these were disproportionately enriched within the CD69^+^CD103^+^ T_RM_ fraction (Fig. 4 g). This supported our hypothesis that CD8 cell-autonomous IL-2 allows liver-resident T cells to survive and maintain functionality. Such maintenance of long-lived antiviral T cells within tissues has been noted in mice after resolution of vesicular stomatitis virus or listeria infection (Masopust et al., 2001). 

In summary, we show for the first time that global and virus-specific CD8 T_RM_ compartmentalized within the vasculature of the healthy human liver expand and persist in patients partially or fully controlling hepatotropic viral infection. These T_RM_ are enriched for liver-homing chemokine receptors and exhibit other adaptations to the liver niche that can be recapitulated using sequential stimulation of PBMCs with IL-15 or TCR engagement and TGFβ. They are distinguished by the combination of high cell-autonomous IL-2 and PD-1 and are poised to mount immediate noncytolytic antiviral effector functions. Our findings have several important implications for current intensive efforts to develop therapeutic vaccines and other immunotherapies for HBV and HCC. Our data underscore the need to sample the liver to assess immunotherapeutic responses by these local, specialized, sentinel T cells, which are not represented in the blood. This is exemplified by a recent study showing that intravenous administration of a malaria vaccine enhanced its efficacy, in association with the expansion of pathogen-specific CD8 T cells in the liver (Ishizuka et al., 2016). We provide a blueprint for the induction of T cells able to reside in the liver, well positioned to maintain hepatic immunosurveillance and exert rapid front-line pathogen defense.

## Materials and methods

### Ethical approval

This study was approved by the local National Health Services Research Ethics Committees for either The Royal London Hospital or The Royal Free Hospital and complies with the Declaration of Helsinki. All healthy controls and patients gave written informed consent before inclusion.

### Samples

Resected liver tissue from the healthy margins of 19 colorectal metastatic tumor resections were obtained through the Tissue Access for Patient Benefit scheme at The Royal Free Hospital (approved by the University College London–Royal Free Hospital BioBank Ethical Review Committee; Research Ethics Committee reference number 11/WA/0077). Additional liver samples were obtained as follows: perfusion liquid (perfusate) from 24 healthy livers used for solid-organ transplantation; tissue from three healthy livers deemed unsuitable for transplantation (e.g., vascular abnormalities, warm ischemic time >30 min, or identification of a tumor elsewhere in the donor); and biopsies taken from eight healthy livers before transplantation (RIPCOLT clinical trial; Research Ethics Committee reference number 11/H0720/4; trial number 8191; trial registered at clinicaltrials.gov: NCT00796588). For comparison, 40 peripheral blood samples from healthy control individuals were included within the study (approved by the South East Coast Research Ethics Committee; Research Ethics Committee reference number 11/LO/0421; IRAS project number, 43993). All healthy control participants used within the study were anti-HBV, anti–hepatitis C, and anti-HIV antibody negative.

For HBV^+^ liver tissue, we used clinical biopsy tissue deemed surplus to diagnostic requirements from 33 patients with treatment-naive CHB, none of whom had end-stage cirrhosis (obtained with paired blood samples from the same individuals), from The Royal London Hospital (approved by East London and The City Research Ethics Committee; Research Ethics Committee reference number P/01/023). We also used two perfusates from HBV-exposed donor livers (one HBsAg^−^, anti-HBsAb^+^, anti-HBcAb^+^ resolved, and one HBsAg^+^ healthy carrier; RIPCOLT trial) and two margins of resected HCCs from The Royal Free Hospital (Tissue Access for Patient Benefit). Participants with chronic HBV infection were anti–hepatitis C and anti-HIV antibody negative, and all were treatment naive (apart from the two from whom resections were obtained) and were stratified by HBV viral load (IU/ml; determined by real-time PCR), serum HBsAg titer (IU/ml; determined by Architect; Abbott Diagnostics), presence of HBeAg, and serum alanine transaminase (IU/liter).

### PBMC and IHL isolation

PBMCs were isolated from heparinized blood by density centrifugation using Ficoll-Hypaque Plus (GE Healthcare). For CD8 T_RM_ quantification, all samples were used immediately where possible. Samples not used immediately were frozen in 10% DMSO (Sigma-Aldrich) in FBS (Sigma-Aldrich) and were stored in accordance with the Human Tissue Act. IHLs from biopsy tissue of either healthy livers or livers from patients with CHB were isolated by mechanical disruption using cell scrapers without further processing (debris removed by passing single cell suspension through 70-µM cell strainers from BD). With larger explant tissues, sections were cut into small pieces and incubated for 30 min at 37°C in 0.01% collagenase IV (Invitrogen) and 0.001% DNase I (Sigma-Aldrich). After enzymatic digestion, mechanical digestion was performed using a GentleMACS (Miltenyi Biotec). After full digestion, debris was removed as before, and parenchymal cells were removed by centrifugation on a 30% Percoll gradient (GE Healthcare). Finally, IHLs were isolated by density centrifugation using Ficoll-Hypaque Plus. When processing perfusate samples, perfusion liquid was first concentrated by centrifugation. Concentrated cells were resuspended in RPMI 1640 (Gibco), and lymphocytes were isolated by density centrifugation using Ficoll-Hypaque Plus.

### Flow cytometry for T_RM_ phenotype and function

Multiparametric flow cytometry was used for phenotypic and functional analysis of PBMCs and IHLs. Cells were stained with a fixable Live/Dead dye (Invitrogen) before incubation with saturating concentrations of surface mAbs diluted in 50% Brilliant violet buffer (BD) and 50% PBS for 30 min at 4°C. See also Table S1 for full details regarding antibodies used. Cells were fixed and permeabilized for further functional assessment with either Cytofix/Cytoperm (BD) or FoxP3 Buffer Set (BD) according to the manufacturer’s instructions. Saturated concentrations of mAbs for 30 min at 4°C were diluted in 0.1% saponin (Sigma-Aldrich) for the detection of intracellular proteins or in 1× PBS for the detection of intranuclear proteins. All samples were acquired on either an LSRII or X20 flow cytometer (BD) and analyzed using FlowJo (Tree Star).

### Induction of T_RM_ phenotype

PBMCs from healthy controls at 3 × 10^5^ cells/well were incubated with either (i) 5, 10, or 50 ng/ml of recombinant human (rh) IL-15, TGFβ, IL-7, IL-33, CXCL16, IL-6, or IL-12 (R&D Systems) or combinations of these for cytokine induction; (ii) 0.25 µg/ml immobilized anti-CD3 (eBioscience) for 24 h and then rested for a further 48 h before increasing doses of rhTGFβ or 5 d resting; or (iii) with 1 µg/ml overlapping peptide (pool of 15-mer peptides overlapping by 10 residues) spanning the core (JPT Technologies), envelope, and polymerase (Massachusetts General Hospital Peptide Synthesis Facility) proteins of the HBV genotype Dor 5 µg/ml HLA-A– and HLA-B–restricted peptide pools spanning the immunodominant proteins of CEF (JPT Technologies) for 7 d. All experiments were done in complete RPMI (cRPMI; RPMI-1640 containing 10% FBS, 100 U/ml penicillin/streptomycin, 1× nonessential amino acids, 1× essential amino acids, and β-mercaptoethanol; Invitrogen) and cultured in the presence of 20 IU/ml rhIL-2 (Miltenyi Biotec) for 3/6/7 d at 37°C. After culture, cells were stained for CD8^+^ T_RM_ frequency, surface phenotype, and transcription factor profiling by flow cytometry as in the prior section. In some experiments, naive (CD27^+^CD45RA^+^) and memory (CD45RA^−^) CD8 T cells were FACS sorted from five different PBMC donors before T_RM_ induction with cytokine or TCR engagement with immobilized anti-CD3.

### Immunohistochemistry/Immunofluorescence

For immunohistochemistry, healthy margin or diseased liver samples from patients with CHB were obtained from surgery, fixed in neutral buffered formalin solution, and embedded in paraffin. 5-µm sections were mounted and rehydrated in xylene, descending alcohol concentrations, and distilled water. For antigen retrieval, slides were microwaved for 8 min in 10 mmol/liter Tris and 1 mmol/liter EDTA buffer, pH 9, and equilibrated in PBS. Endogenous peroxidase activity was quenched by immersion in 1% H_2_O_2_/MeOH for 10 min. Nonspecific antibody binding was blocked with 5% nonfat skimmed milk in PBS for 30 min, and slides were incubated in primary antibody diluted in 1% BSA in PBS overnight at 4°C. After washing in PBS, slides were immersed for 30 min in the corresponding ImmPRESS HRP Polymer Detection solution (Vector Laboratories). Antibody complexes were visualized by brief incubation in chromagen DAB/SG HRP substrate (Vector Laboratories). Slides were rinsed in distilled water, counterstained in hematoxylin, dehydrated via an ascending alcohol gradient and xylene, and mounted in a mixture of distyrene, plasticizer, and xylene. For immunofluorescence, frozen healthy liver margins from surgery or core needle–biopsy specimens obtained from CHB patients for diagnostic purposes were embedded in optimum cutting temperature compound (RA Lamb) and frozen on dry ice. 5-µm sections were cut, mounted, fixed in acetone for 10 min and then were air dried and washed in PBS. Nonspecific antibody binding was blocked with 2.5% normal horse serum (Vector Laboratories) for 30 min before incubation with primary antibody diluted in 1% BSA in PBS overnight at 4°C. After washing with PBS, slides were incubated in the appropriate secondary antibody diluted in 1% BSA in PBS for 1 h at room temperature and then were washed and mounted in Vectashield mounting medium with DAPI (Vector Laboratories). Sections were imaged using a high-resolution digital Axio Scan.Z1 slide scanner and associated Zen software (ZEISS). Primary and secondary antibodies used were as follows: mouse anti–human e-cadherin (HECD-1; Abcam), anti–human cytokeratin-FITC (CK3-6H5; Miltenyi Biotech), rat anti–human CD8 (YTC141.1HL; AbD Serotec), mouse anti–human CD69 (FN50; BioLegend), mouse anti–human CD8 (C8/144B; Dako), rabbit anti–human integrin α E (CD103; EPR4166(2); Abcam), rabbit anti–human PD-L1 (E1L3N; Cell Signaling Technology), anti–mouse IgG1 Alexa Fluor 568 (Thermo Fisher Scientific), anti–rat IgG Alexa Fluor 488 (Thermo Fisher Scientific), anti–mouse IgG1 Alexa Fluor 488 (Thermo Fisher Scientific), and anti–rabbit IgG Alexa Fluor 594 (Thermo Fisher Scientific).

### Dextramer staining for the identification of virus-specific T_RM_

HBV-specific HLA-A2 dextramers (Immudex) of the following specificities were used: core 18–27 (FLPSDFFPFV), envelope 183–191 (FLLTRILTI), envelope 335–342 (WLSLLVPFV), envelope 348–357 (GLSPTVWLSV), polymerase 455–463 (GLSRYVARL), and polymerase 502–510 (KLHLYSHPI). Cells were stained with dextramers at 37°C in 1× PBS, washed twice in cRPMI, and left to rest for 1 h in cRPMI before further mAb staining as described in the Flow cytometry for T_RM_ phenotype and function section (Fig. 4, b and f). During analysis, stringent gating criteria were applied with doublet, dead, and CD19^+^ cell exclusion to minimize nonspecific binding contamination. A dextramer loaded with an irrelevant peptide was used in parallel to control for nonspecific binding.

### Functional assessment of CD8 T_RM_

To assess the production of cytokines by CD8 T_RM_, 10^6^ IHLs were stimulated with either 0.5 µg/ml immobilized anti-CD3 and 5 µg/ml anti-CD28 (eBioscience); 10 µg/ml overlapping peptides (pool of 15-mer peptides overlapping by 10 residues) spanning the core, envelope, and polymerase proteins of HBV genotype D; or 5 µg/ml HLA-A– and HLA-B–restricted peptide pools spanning the immunodominant proteins of CEF. For HLA-A2^+^ donors, IHLs were stimulated with 10 µM HBV-derived HLA-A2 restricted peptides (FLPSDFFPSV [core]; FLLTRILTI, WLSLLVPFV, LLVPFVQWFV, and GLSPTVWLSV [envelope]; and GLSRYVARL and KLHLYSHPI [polymerase]; ProImmune). All functional experiments were performed in the presence of 1 µg/ml brefeldin-A (Sigma-Aldrich) for 4 h or 16 h at 37°C. Specific responses were detected by intracellular cytokine staining as described in the Flow cytometry for T_RM_ phenotype and function section (Figs. 1 and 4).

### Statistical analysis

Statistical analyses were performed in either Prism (GraphPad Software) or R version 3.2.4 using appropriate methods as indicated in the legends (Mann-Whitney *t* test, Wilcoxon Signed-rank *t* Test, Kruskal-Wallis test [ANOVA] with a Dunn’s post hoc test for pairwise multiple comparisons between each group, Spearman’s Rank Order Correlation, or multivariate ANOVA [MANOVA]), with significant differences marked on all figures. Specifically, the MANOVA was used when more than one dependent variable of interest was considered simultaneously to more than one independent variable of interest. Where appropriate, the Bonferroni correction method for multiple testing was used. All tests were performed as two-tailed tests, and for all tests, significance levels were defined as *, P < 0.05; **, P < 0.01; ***, P < 0.001; and ****, P < 0.0001.

### tSNE analysis

tSNE analysis was performed on flow cytometry data from four perfusates stained on cytobank as described in the Flow cytometry for T_RM_ phenotype and function section (Kotecha et al., 2010) using default parameters (Iterations, 1,000; perplexity, 30; and θ, 0.5). tSNE was applied to expression data for CD69, CD103, Blimp-1, Eomes, and T-bet for all live CD3^+^CD8^+^CD45RA^−^ events after concatenating four donor perfusates.

### Online supplemental material

Fig. S1 shows a profile of liver-resident memory CD8 T cell in the healthy human liver. Fig. S2 shows the function and phenotype of T_RM_. Fig. S3 shows the phenotypic and transcription profiling of CD45RA^−^CD69^+^CD103^−^ and CD69^+^CD103^+^ intrahepatic CD8 T cells. Table S1 is a list of monoclonal antibody details.

## Supplementary Material

Supplemental Materials (PDF)
