# Supplementary material for: IL-2high tissue-resident T cells in the human liver: Sentinels for hepatotropic infection
Source: J Exp Med. 2017 Jun 5;214(6):1567–80. doi: 10.1084/jem.20162115 (PMC5461007; doi:10.1084/jem.20162115)
Supplement: Supplemental Materials (PDF) [file JEM_20162115_sm.pdf]

The Journal of Experimental Medicine

## SUPPLEMENTAL MATERIAL

Pallett et al., https://doi.org/10.1084/jem.20162115

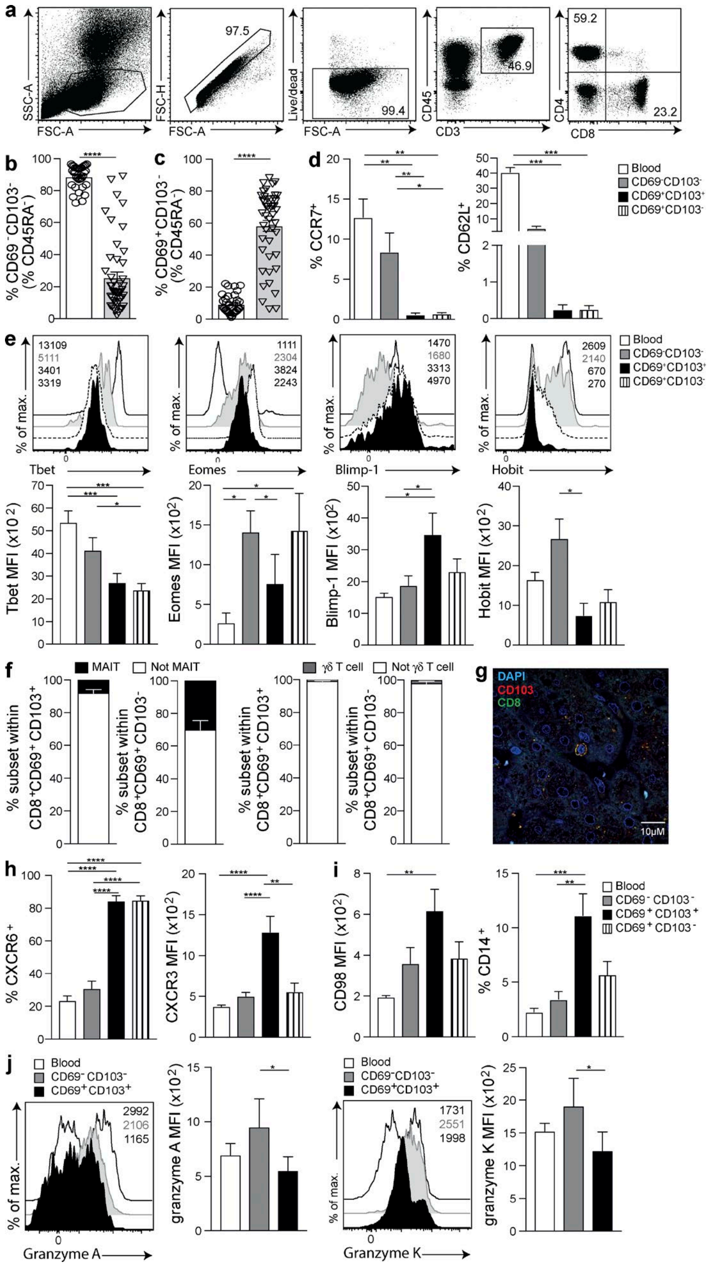

Figure S1. **Profile of liver-resident memory CD8 T cell in the healthy human liver.** (a) Boxes on FACS plots define the representative sequential gating strategy identifying CD8 T cells in the human liver (live lymphocytes, singlets, CD45$^+$CD3$^+$, and CD8$^+$) using 16-color flow cytometry. FSC-A, forward scatter; Live/dead, fixable dead cell stain; SSC-A, side scatter. (b and c) Frequency of the percent CD69$^-$CD103$^-$ (b) and percent CD69$^+$CD103$^-$ (c) within memory (CD45RA$^-$) CD8 in healthy control PBMCs (white; $n$ = 40) or healthy livers (gray; $n$ = 54). (d and e) Summary data from circulating (white), intrahepatic CD45RA$^-$CD69$^-$CD103$^-$ (gray), intrahepatic CD45RA$^-$CD69$^+$CD103$^+$ (black), or intrahepatic CD45RA$^-$CD69$^+$CD103$^-$ (striped bars) memory CD8 T cells from healthy donors for CCR7 (%; $n$ = 7) and CD62L (%; $n$ = 7; d) and T-bet (MFI; $n$ = 22), Eomes (MFI; $n$ = 12), Blimp-1 (MFI; $n$ = 10) and Hobit (MFI; $n$ = 7; e). (f) Frequency of either CD8$^+$CD45RA$^-$CD69$^+$CD103$^+$ or CD8$^+$CD45RA$^-$CD69$^+$CD103$^-$ within a gate for mucosal-associated invariant T cells (MAIT, CD45$^+$CD3$^+$CD8$^+$CD161$^{high}$V$\alpha$7.2$^+$; black) or $\gamma\delta$ T cells (CD45$^+$CD3$^+$pan-$\gamma\delta$TCR$^+$; gray). (g) Immunofluorescence staining of a frozen liver section: CD8 (green) and CD103 (red) showing a representative CD8$^+$CD103$^+$ T cell localized within a sinusoidal space surrounded by hepatocytes. (h and i) Summary data from circulating (white), intrahepatic CD45RA$^-$CD69$^-$CD103$^-$ (gray), intrahepatic CD45RA$^-$CD69$^+$CD103$^+$ (black), or intrahepatic CD45RA$^-$CD69$^+$CD103$^-$ (striped bars) memory CD8 T cells from healthy donors for CXCR6 (%; $n$ = 24), CXCR3 (MFI; $n$ = 21; h), CD98 (MFI; $n$ = 12), and CD14 (%; $n$ = 20; i). (j) Representative histograms and summary data from circulating (white), intrahepatic CD45RA$^-$CD69$^-$CD103$^-$ (gray), and intrahepatic CD45RA$^-$CD69$^+$CD103$^+$ (black) memory CD8 T cells from healthy donors for granzyme A (MFI; $n$ = 12) or granzyme K (MFI; $n$ = 12). Error bars indicate means ± SEM; *, P < 0.05; **, P < 0.01; ***, P < 0.001; ****, P < 0.0001; p-values were determined by a Mann-Whitney $t$ test (b and c) or a Kruskal-Wallis test (ANOVA) with a Dunn's post-hoc test for pairwise multiple comparisons (d, e, h, i, and j).

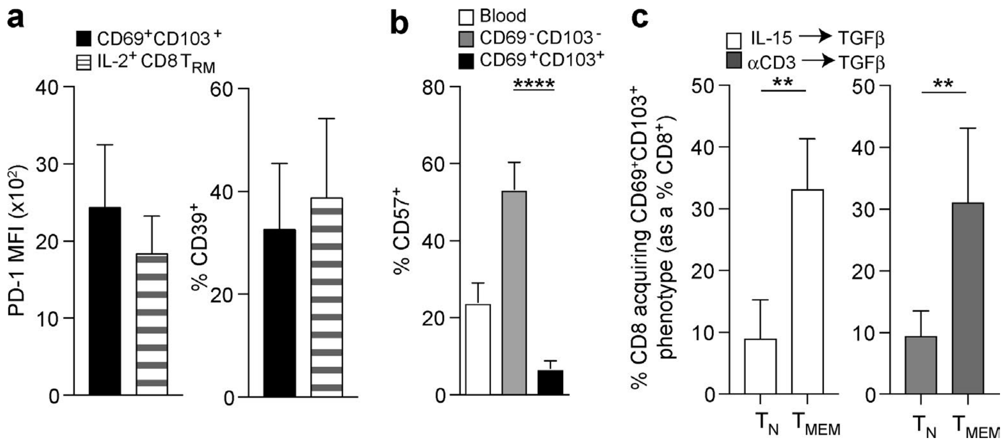


Figure S2. **Function and phenotype of $T_{RM}$.** (a) Expression of PD-1 (MFI; *n* = 9) and CD39 (%; *n* = 4) on global CD8 $T_{RM}$ compared with IL-2$^+$ CD8 $T_{RM}$ (4 h anti-CD3 and anti-CD28). (b) Summary data of CD57 expression (%; *n* = 10) in circulating (white), intrahepatic CD45RA$^-$CD69$^-$CD103$^-$ (gray), and intrahepatic CD45RA$^-$CD69$^+$CD103$^+$ $T_{RM}$ (black) memory CD8 T cells from healthy donors. (c) Frequency of induced CD8 $T_{RM}$ after culture from FACS-sorted naive (CD27$^+$CD45RA$^+$) or memory (CD45RA$^-$) CD8 T cells after sequential exposure to 3 d 50 ng/ml rhIL-15 followed by 3 d 50 ng/ml rhTGFβ or 3 d 0.25 µg/ml immobilized anti-CD3 followed by 3 d 50 ng/ml rhTGFβ (*n* = 5). Error bars indicate means ± SEM; **, $P < 0.01$; ****, $P < 0.0001$; p-values were determined by a Wilcoxon Signed-rank *t* Test (a), a Kruskal-Wallis test (ANOVA) with a Dunn's post-hoc test for pairwise multiple comparisons (b), or a Mann-Whitney *t* test (c).

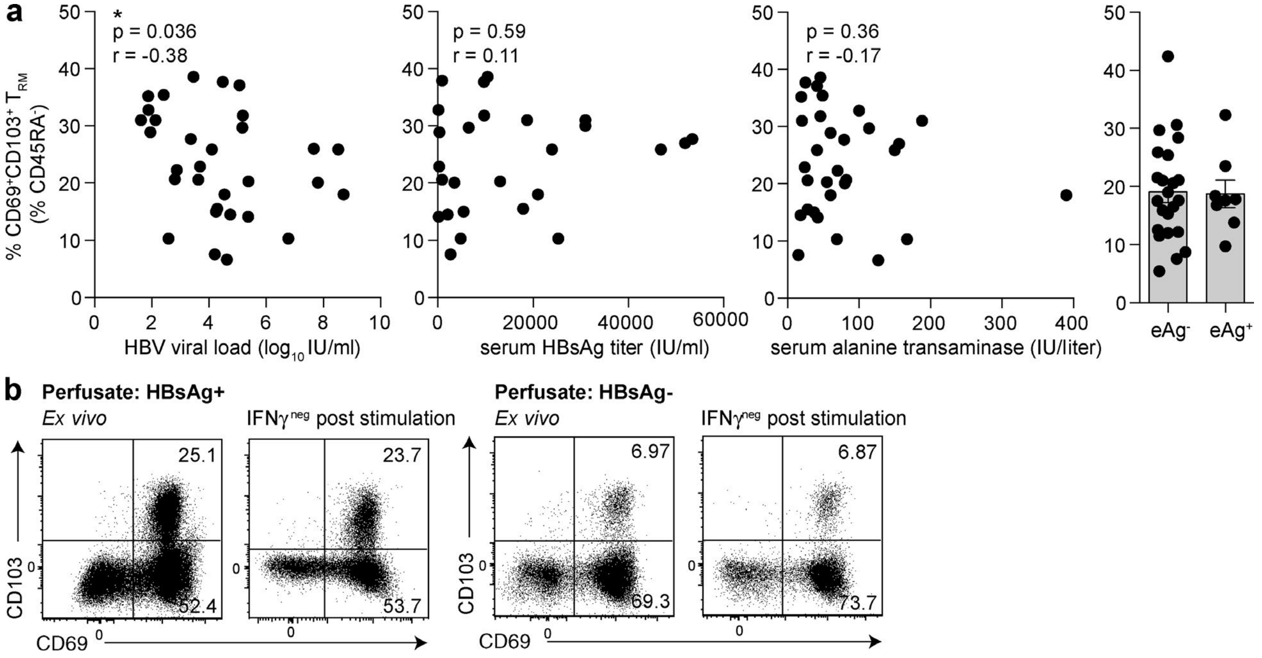


Figure S3. **Phenotypic and transcription profiling of CD45RA$^-$CD69$^+$CD103$^-$ and CD69$^+$CD103$^+$ intrahepatic CD8 T cells.** (a) Frequencies of CD8 $T_{RM}$ (CD45RA$^-$CD69$^+$CD103$^+$) in HBV-infected livers from treatment-naive patients stratified by available clinical parameters: viral load (IU/ml; *n* = 33), serum HBsAg titer (IU/ml; *n* = 27), serum alanine transaminase levels (IU/liter; *n* = 31), and the presence or absence of HBeAg (eAg$^+$, *n* = 8; eAg$^-$, *n* = 23). (b) Representative FACS plots of ex vivo and IFNγ-negative CD8 T cells for CD69 and CD103 expression from the chronic (HBsAg$^+$) and resolved (HBsAg$^-$) HBV perfusates used for peptide stimulations in Fig. 4 (c and g). Error bars indicate means ± SEM; *, $P < 0.05$; p-values were determined by Spearman's Rank Order Correlation or Mann-Whitney *t* test.

Table S1. **Monoclonal antibody details**

| Antigen | Fluorochrome | Manufacturer | Clone | Catalog number |
|---|---|---|---|---|
| **Phenotype** | | | | |
| CD45 | BUV805 | BD | HI30 | 564914 |
| CD3 | BV711 | BioLegend | OKT3 | 317328 |
| CD8a | Alexa-Fluor700 | eBioscience | OKT8 | 56-0086-82 |
| CD4 | APC/Cy7 | BD | RPA-T4 | 557871 |
| CD4 | BV421 | BD | RPA-T4 | 562425 |
| CD27 | BUV395 | BD | LI28 | 563815 |
| CD45RA | PE/Cy7 | BioLegend | HI100 | 304126 |
| CD45RA | eFluor450 | eBioscience | HI100 | 48-0458-41 |
| CD69 | PE/Dazzle 594 | BioLegend | FN50 | 310942 |
| CD69 | BV605 | BioLegend | FN50 | 310937 |
| CD103 | BV605 | BioLegend | Ber-ACT8 | 350218 |
| CD103 | FITC | BioLegend | Ber-ACT8 | 350203 |
| HLA-DR | Horizon V500 | BD | G46-6 | 561224 |
| CD39 | BV421 | BioLegend | A1 | 328214 |
| CXCR6 | APC | BioLegend | K041E5 | 356006 |
| CXCR3 | PerCP-Cy5.5 | BD | IC6 | 560832 |
| CCR7 | PE/Cy7 | BD | 3D12 | 557648 |
| CD62L | APC/Cy7 | BioLegend | DREG-56 | 304813 |
| PD-1 | PE | BioLegend | EH12.2H7 | 329906 |
| CD98 | FITC | BioLegend | MEM-108 | 315603 |
| CD14 | Horizon V500 | BD | M5E2 | 561391 |
| CD57 | eFluor450 | eBioscience | TB0 | 48-0577-42 |
| CD19 | Horizon V510 | BD | 5J25CI | 562947 |
| CD161 | APC | Miltenyi Biotec | 191B8 | 130-098-908 |
| Vα7.2 | FITC | BioLegend | 3C10 | 351704 |
| γδ-TCR | APC | BioLegend | B1 | 331211 |
| **Function** | | | | |
| IFNγ | Horizon V450 | BD | B27 | 560371 |
| IL-2 | PerCP-eFluro710 | eBioscience | MQ1-17H12 | 46-7029-42 |
| TNF | FITC | BD | MAb11 | 554512 |
| Perforin | PerCP-Cy5.5 | eBioscience | dG9 | 308113 |
| Granzyme B | FITC | BioLegend | GB11 | 515403 |
| Granzyme A | PE | BioLegend | CB9 | 507206 |
| Granzyme K | Alexa Fluor 647 | BioLegend | CM26E7 | 370503 |
| Ki67 | FITC | BD | B56 | 556026 |
| **Transcriptional profiling** | | | | |
| T-bet | eFluor 660 | eBioscience | eBio4B10 | 50-5825-82 |
| Eomes | PE eFluor-610 | eBioscience | WD1928 | 61-4877-41 |
| Notch | BV421 | BD | MHN1-519 | 564783 |
| Blimp-1 | PE | BD | 6D3 | 564702 |
| Hobit | Unconjugated | A gift from Klaas van Gisbergen (Sanquin) | | |
| Anti-IgM | FITC | BD | DS-1 | 553516 |
